# Transmittance of UVB, UVA, and visible light (blue-violet) among the main Brazilian commercial opaque sunscreens^☆^^☆☆^

**DOI:** 10.1016/j.abd.2019.01.004

**Published:** 2019-10-24

**Authors:** Gabriel Peres, Hélio Amante Miot

**Affiliations:** a Postgraduate Program in Surgical Foundations, Faculdade de Medicina, Universidade Estadual Paulista, Botucatu, SP, Brazil; b Department of Dermatology and Radiotherapy, Faculdade de Medicina, Universidade Estadual Paulista, Botucatu, SP, Brazil

*Dear Editor*,

Photoprotection is essential in the prevention and treatment of photo-induced dermatoses. Environmental and geographic factors should be weighed in the indication of the photoprotection strategy, such as sunscreens (SSs), mechanical photoprotection (coverings, glasses, clothing), and behavioral aspects.^1^

SSs use compounds that interfere with the penetration of solar radiation into the skin, minimizing its biological tissue effects. Such substances can be organic or inorganic, and pigments are used to potentiate visible light (VL) blockage.^1^, ^2^

As different types of radiation trigger characteristic pathological processes, knowledge of SS photoprotection patterns is essential for their indication. The prevention of sunburn is associated with the sun protection factor (SPF), and the persistent pigmentation prevention is associated with persistent pigment darkening (PPD) and the maintenance of immunological surveillance of the skin by the immune protection factor.^1^, ^3^

The VL spectrum (400–780 nm) is below the UVA range, and represents 40% of the incident solar energy, which can promote persistent pigmentation in higher phototypes and free radicals in the stratum corneum. However, VL promotes less tissue interaction and its effects are dozens of times less intense than those promoted by UVA and thousands of times smaller than those induced by UVB.^1^, ^4^

The most biologically active fraction of VL comprises the blue-violet range (400–500 nm), and may be relevant in preventing aging and dyschromia, such as melasma, as well as UVA. However, there is still no universally accepted method to evaluate the photobiological protection against VL, nor any reference to this protection in the SS.^5^

Topical protection against VL is promoted by opaque SS. In Brazil, there are SSs marketed with a proposed VL block; however, there is no clear picture of the simultaneous protections against the other radiation ranges offered by these products.

A cross-sectional study was conducted to evaluate the *in vitro* transmittance of UVB, UVA, and blue-violet light (400–500 nm) among the major Brazilian commercial SSs. There were 41 opaque SSs with SPF > 30 evaluated between September 2017 and September 2018. The characteristics of the SS tested are provided in Table 1.

**Table 1:** Main characteristics of the 44 commercial sunscreens tested.

Product/brand	Manufacturer	Lot	SPF	UVA
Actsun Color FPS 60	FQM	171099	50	VHP
Anthelios Airlicium FPS 70 – clear	La Roche Posay	58R17M	70	VHP
Anthelios Airlicium FPS 70 – with color	La Roche Posay	60p2e	70	VHP
Anthelios Airlicium FPS 70 – morena	La Roche Posay	58R17M	70	VHP
Anthelios Airlicium FPS 70 – morena mais	La Roche Posay	58R1EM	70	VHP
Anthelios Alta cobertura FPS 60	La Roche Posay	6ON3F	60	VHP
Anthelios BB cream FPS 50	La Roche Posay	3605054	50	HP/28 ≤ 2.5^a^
Blocskin FPS 40 Color	Vitalife	12004171	40	+
Cetaphil Sun FPS 70 – with color	Galderma	14190118	70	VHP
Emulsão Color FPS 70	Avène	1700181	70	VHP
Emulsão Color FPS 50+	Avène	av196	50	VHP
Ensoleil Extreme FPS 90+	Ache Profuse	L1513664	90	VHP/31^b^
Episol color FPS 70 – pele clara	Mantecorp	b17b2203	70	VHP
Episol color FPS 70 – pele extra clara	Mantecorp	B18E1465	70	VHP
Episol color FPS 70 – pele morena	Mantecorp	B16M1847	70	VHP
Episol color FPS 70 – pele morena mais	Mantecorp	B18F1906	70	VHP
Episol color FPS 70 – pele negra	Mantecorp	B18E1464	70	VHP
Filtrum Color FPS 50	Libbs	1701001a	50	19^a^
Foto Ultra Active Unify Fusion Fluid color FPS 99	Isdin	50901	99	VHP/49^a^
Fotoprotector Gel Cream Dry Touch Color FPS 50+	Isdin	3296100	60	VHP
FQM-Melora Heliocare® Gel Color Nude Bronze FPS 50	FQM	16L245	50	+
Idéal Capital Soleil FPS 50 – com cor	Vichy	60p2b	50	HP
Idéal Soleil Clarify FPS 60 – clara	Vichy	58R77M	60	VHP
Idéal Soleil Clarify FPS 60 – media	Vichy	58R79M	60	VHP
Idéal Soleil Clarify FPS 60 – morena	Vichy	58R79M	60	VHP
Idéal Soleil Clarify FPS 60 – com cor	Vichy	60P801	60	VHP
Minesol Actif Unify FPS 60 – light	ROC	1957B01	60	VHP
Minesol Actif Unify FPS 60 – medium	ROC	3486B01	60	VHP
Minesol Oil Control FPS 60 – tinted gel creme universal	ROC	2566k	60	VHP
Moderm Protetor Solar com base FPS 35 – bege claro	Galderma	1651	35	VHP
Moderm Protetor Solar com base FPS 35 – bege médio	Galderma	1651	35	VHP
Photoderm M FPS 50+	Bioderma	11761	70	VHP/36^c^
Photoderm Max Nude Touch FPS 50+ – claro	Bioderma	N1X85881Q607V	50	VHP/25^c^
Photoderm Max Nude Touch FPS 50+ – dourado	Bioderma	N1X85891Q607V	50	VHP/25^c^
Photoderm Max Nude Touch FPS 50+ muito claro	Bioderma	N1X85871Q607V	50	VHP/25^c^
Photoderm Max Toque Seco FPS 60 – Tinto	Bioderma	33651	60	VHP/37^b^
Photoderm Max Toque Seco FPS 90 – Tinto	Bioderma	2961	90	VHP/38^b^
Photoprot FPS 99 Color	Biolab	1009319	99	VHP/62^a^
Physical Matte UV defense FPS 50	SkinCeuticals	jcp33w	50	HP
Eucerin Sun creme tinted FPS 60	Eucerin	L6226034	60	VHP
Sunfresh facial com cor FPS 60	Neutrogena	1377B01	60	+

*Negative controls*				
Anthelios XL Protect FPS 70	La Roche Posay	60n7tc3	70	VHP
Eryfotona AK-NMSC Fluid	Isdin	53461	99	NA
FotoUltra – Spot Prevent – Fusion Fluid 99	Isdin	5057a	99	VHP/61^a^

UVA, ultraviolet A; PPD, persistent pigment darkening; FPS, sun protection factor; NA, not available; +, only mentioned “UVA”; HP, high protection; VHP, very high protection.

a UVA protection factor.

b PPD.

c UVA method not mentioned.

Samples of 500 mg of each product were dispersed in 250 cm^2^ of transparent film, in order to reach 2 mg/cm^2^, and submitted to artificial sources of UVB (230 μW/cm^2^), UVA (1270 μW/cm^2^), and blue-violet VL (400–520 nm, 729 mW/cm^2^). The values of transmittance were evaluated by the following apparatuses: UVB Digital Ultraviolet Radiometer (ZooMed, San Luis Ubispo, CA, United States), Digital Ultraviolet Radiometer 4.2 UVA (Solarmeter, Glenside, PA, United States), and Radiometer RD-7 (Ecel, Ribeirão Preto, SP, Brazil).

Additionally, three pigment-free SSs were evaluated as controls of the experiment (Table 1).

The calculated transmittance was the percentage of radiation that passes through each SS, being complementary to the value of the sum of the absorbance. For its calculation, multiple measurements were taken on the surface covered with SS, and the mean value was calculated, divided by the irradiation of each source through the transparent film, without SS.

All SS tested showed UVB transmittance <0.1%. The UVA and VL transmittances are shown in Table 2. In general, opaque SSs had higher UVA coverage than the controls. It is noteworthy that, of the opaque SSs, 63% (26/41) blocked >99.9% of UVA and 63% (26/41) blocked >99.9% of blue-violet light. However, this blockade was not concurrent, since 31% (8/26) of the opaque SSs that blocked >99.9% of the VL did not have the same performance for the UVA.

**Table 2:** Percentage of ultraviolet A (UVA) and visible light transmittance of the different sunscreens tested (*n* = 44).

Product/brand	UVA	LV
Actsun Color FPS 60	0.0%	6.0%
Anthelios Airlicium FPS 70 – clara	0.0%	0.0%
Anthelios Airlicium FPS 70 – com cor	0.0%	0.0%
Anthelios Airlicium FPS 70 – morena	0.0%	0.0%
Anthelios Airlicium FPS 70 – morena mais	0.0%	0.0%
Anthelios Alta cobertura FPS 60	0.0%	0.0%
Anthelios BB cream FPS 50	0.4%	7.4%
BLOCSKiN FPS 40 color	0.0%	6.8%
Cetaphil Sun FPS 70 – com cor	0.1%	0.0%
Emulsão Color FPS 70	0.1%	16.7%
Emulsão Color FPS 50+	0.2%	6.8%
Ensolei Extreme FPS 90+	0.1%	4.7%
Episol color FPS 70 – pele clara	0.0%	0.0%
Episol color FPS 70 – pele extra clara	0.0%	0.0%
Episol color FPS 70 – pele morena	0.0%	0.0%
Episol color FPS 70 – pele morena mais	0.0%	0.0%
Episol color FPS 70 – pele negra	0.0%	0.0%
Eucerin Sun Creme tinted FPS 60	0.0%	11.4%
Filtrum Color FPS 50	0.0%	0.0%
Foto Ultra Age Active Unify Fusion Fluid color FPS 99	0.0%	0.0%
Fotoprotector Gel Cream Dry Touch color FPS 50+	0.0%	3.2%
FQM-Melora Heliocare® gel color nude bronze FPS 50	0.1%	3.1%
Idéal Capital Soleil FPS 50	0.0%	0.0%
Idéal Soleil Clarify FPS 60 – clara	0.0%	0.0%
Idéal Soleil Clarify FPS 60 – média	0.0%	0.0%
Idéal Soleil Clarify FPS 60 – morena	0.0%	0.0%
Idéal Soleil Clarify FPS 60 com cor	0.0%	0.0%
Minesol Actif Unify FPS 60 – light	0.1%	0.0%
Minesol Actif Unify FPS 60 – medium	0.4%	0.0%
Minesol Oil Control FPS 60 tinted	0.0%	17.7%
Moderm Protetor Solar com base FPS 35 – bege claro	0.1%	0.0%
Moderm Protetor Solar com base FPS 35 – bege médio	0.1%	0.0%
Photoderm M FPS 50+	0.0%	7.1%
Photoderm MAX Nude Touch FPS 50+– claro	0.5%	0.0%
Photoderm MAX Nude Touch FPS 50+– dourado	0.8%	0.0%
Photoderm MAX Nude Touch FPS 50+– muito claro	1.2%	0.0%
Photoderm MAX Toque Seco FPS 60 Tinto	0.0%	3.9%
Photoderm MAX Toque Seco FPS 90 Tinto	0.0%	0.0%
Photoprot FPS 99 Color	0.1%	3.9%
Physical Matte UV defense FPS 50	0.9%	6.8%
Sunfresh facial com cor FPS 60	0.0%	26.2%

*Negative controls*		
Anthelios XL Protect FPS 70	0.0%	75.4%
Eryfotona AK-NMSC Fluid FPS 99	0.0%	59.5%
FotoUltra – Spot Prevent – Fusion Fluid 99	0.0%	63.4%

*Note*: UVB transmittance <0.1% for all tested products.

Opaque SSs marketed in Brazil showed great variability in UVA and VL transmittance, despite excellent UVB performance. Interestingly, opaque SSs of the same commercial line, but of different shades, did not demonstrate differences in transmittance of VL.

It was observed that 73% (30/41) of the opaque products had no UVA-related values on the label; 7.3% (3/41) used UVAPF and only 7.3% (3/41), PPD.

The transmittance of SS is not perfectly parallel to the biological effect of radiation; however, it is a reasonable way to compare SS performance *in vitro*. Even the critical wavelength, an indicator of UVA protection, is based on the transmittance curve within the UVA spectrum.^1^

Moreover, the biological effect of UVA and VL can be reduced by the use of adjuncts such as antioxidants, present in several SSs tested.

Our results do not deprecate the studied opaque SSs, but highlight their intrinsic differences. These results should be confirmed with *in vivo* methodologies.

In conclusion, SS labels should provide detailed information on SPF and PPD (or another UVA standard) to favor the indication of SS in different clinical settings. This is especially relevant in pigmented dermatoses, more sensitive to UVA, because different opaque SSs with good performance against VL allow a significant passage of UVA, albeit inferior to the transparent SSs tested.

## Financial support

None declared.

## Author's contribution

Gabriel Peres: Approval of the final version of the manuscript; elaboration and writing of the manuscript; obtaining, analyzing and interpreting the data; critical review of the literature.

Hélio Amante Miot: Approval of the final version of the manuscript; conception and planning of the study; effective participation in research orientation; critical review of the literature; critical review of the manuscript

## Conflicts of interest

None declared.
